# Implementation of a software-based decision support tool for guideline-appropriate preoperative evaluation: a prospective agreement study

**DOI:** 10.1016/j.bja.2024.06.001

**Published:** 2024-07-05

**Authors:** Simone M. Kagerbauer, Jennifer Wißler, Dimislav I. Andonov, Bernhard Ulm, Gerhard Schneider, Armin H. Podtschaske, Manfred Blobner, Bettina Jungwirth

**Affiliations:** 1 Department of Anaesthesiology and Intensive Care Medicine, School of Medicine, Technical University of Munich, Munich, Germany; 2 Department of Anaesthesiology and Intensive Care Medicine, School of Medicine, University of Ulm, Ulm, Germany

**Keywords:** digital guideline support, elective noncardiac surgery, guideline adherence, preoperative evaluation, software-based decision-support

## Abstract

**Background:**

Guideline adherence in the medical field leaves room for improvement. Digitalised decision support helps improve compliance. However, the complex nature of the guidelines makes implementation in clinical practice difficult.

**Methods:**

This single-centre prospective study included 204 adult ASA physical status 3–4 patients undergoing elective noncardiac surgery at a German university hospital. Agreement of clearance for surgery between a guideline expert and a digital guideline support tool was investigated. The decision made by the on-duty anaesthetists (standard approach) was assessed for agreement with the expert in a cross-over design. The main outcome was the level of agreement between digital guideline support and the expert.

**Results:**

The digital guideline support approach cleared 18.1% of the patients for surgery, the standard approach cleared 74.0%, and the expert approach cleared 47.5%. Agreement of the expert decision with digital guideline support (66.7%) and the standard approach (67.6%) was fair (Cohen's kappa 0.37 [interquartile range 0.26–0.48] *vs* 0.31 [0.21–0.42], *P*=0.6). Taking the expert decision as a benchmark, correct clearance using digital guideline support was 50.5%, and correct clearance using the standard approach was 44.6%. Digital guideline support incorrectly asked for additional examinations in 31.4% of the patients, whereas the standard approach did not consider conditions that would have justified additional examinations before surgery in 29.4%.

**Conclusions:**

Strict guideline adherence for clearance for surgery through digitalised decision support inadequately considered patients, clinical context. Vague formulations, weak recommendations, and low-quality evidence complicate guideline translation into explicit rules.

**Clinical trial registration:**

NCT04058769.

**Editor's key points**

• Guidelines are developed by professional organisations in the hope that they will be used to standardise preoperative evaluation, optimise risk assessment, and improve patient outcomes.
• In this prospective agreement study conducted at a German academic centre, high-risk adult patients were evaluated by supervised trainee anaesthetists in the preoperative clinic (standard approach) and by specially trained anaesthetists using a digital guideline support tool. The clearance evaluations of each were assessed by a single guideline expert. The primary outcome was the decision to clear for surgery.
• The digital guideline support approach cleared 18.1% of the patients for surgery, the standard approach cleared 74.0%, and the expert cleared 47.5%. Agreement of the expert's decision with digital guideline support and with the standard approach was only fair. The digital guideline support approach tended to be over-cautious with respect to following guidelines, whereas the standard approach tended to fail to follow guidelines.
• Guidelines offer some recommendations that are strongly supported by evidence and others that are weakly supported or are not feasible or appropriate in individual patients. In addition to taking these factors into account, experts are able to incorporate surgery-related factors in risk evaluation, such as expected blood loss.
• Although this study did not prove that the digital guideline support tool in use improved practice, it does stimulate more reflection on whether and how guidelines should be implemented in practice.

Despite constantly improving anaesthesia and surgical techniques, postoperative mortality is still high.^1^ To determine the individual risk, tailor perioperative care, and optimise preoperative status, patients are evaluated by an anaesthetist before elective surgery. To standardise the preoperative evaluation, numerous medical societies issue guidelines.^2^, ^3^, ^4^ Although most physicians report knowledge of these guidelines, their integration into clinical routine needs to be improved.^5^^,^^6^ For instance, although the authors' hospital had implemented and trained staff on the current European and German guidelines for preoperative evaluation, adherence was insufficient, including recommendations that were not followed (up to 53%) as well as unnecessarily ordered examinations (67%, experience from our department). Variance between different guidelines and discrepancy of simultaneously applicable guidelines covering the same topic leads to uncertainty among clinicians. Furthermore, the complexity of guidelines, with their numerous cross-references to guidelines of other professional societies, and the large number of recommendations make it almost impossible for physicians to memorise and consequently comply. Digitalised decision support could provide physicians with the necessary information to optimise adherence to guidelines and thereby reduce complications.^7^

The guideline-based digital decision support tool we used in this study is an independent software module for pre-anaesthesia data management of the QCare patient data management system family (HIM™ Health Information Management GmbH, Bad Homburg, Germany) designed to assist guideline adherence for preoperative evaluation, supported by the innovation programme for small and medium-sized enterprises of the German Federal Ministry for Economic Affairs and Energy (ZF4544701TS8). The software in the investigated version is a demonstrator that can form the basis for a medical device. Recommendations for the preoperative evaluation of adult patients undergoing noncardiac surgery were adopted from the guidelines of the European Society of Anaesthesiology and Intensive Care Medicine (ESAIC),^2^ the European Society of Cardiology (ESC),^4^ and the German Society of Anaesthesiology and Intensive Care Medicine (DGAI).^3^ To collect all the information required by the guidelines in a structured, user-friendly, and fast manner, the developers of the software have taken some conceptual specifications into account: (1) recommendations that are controversial between guidelines have been included by consensus of three experts; (2) recommendations are operationalised according to Shiffman^8^; (3) recommendations are broken down into dichotomous decision trees or logical AND/OR links (Fig. 1); (4) responses include only dichotomous, nominal, or numeric input options (i.e. free text is rigorously avoided); (5) findings are hierarchically assigned to structured organ systems; (6) findings in subordinate specifications are only queried in case of positive findings in the superordinate hierarchy; and (7) responses required to verify the relevance of certain recommendations are mandatory fields that either exist *a priori* or result from upstream data (e.g. a detailed cardiac history is only mandatory if cardiac risk factors are given). An example of the user interface is shown in Supplementary Figure S1.

**Fig 1:**
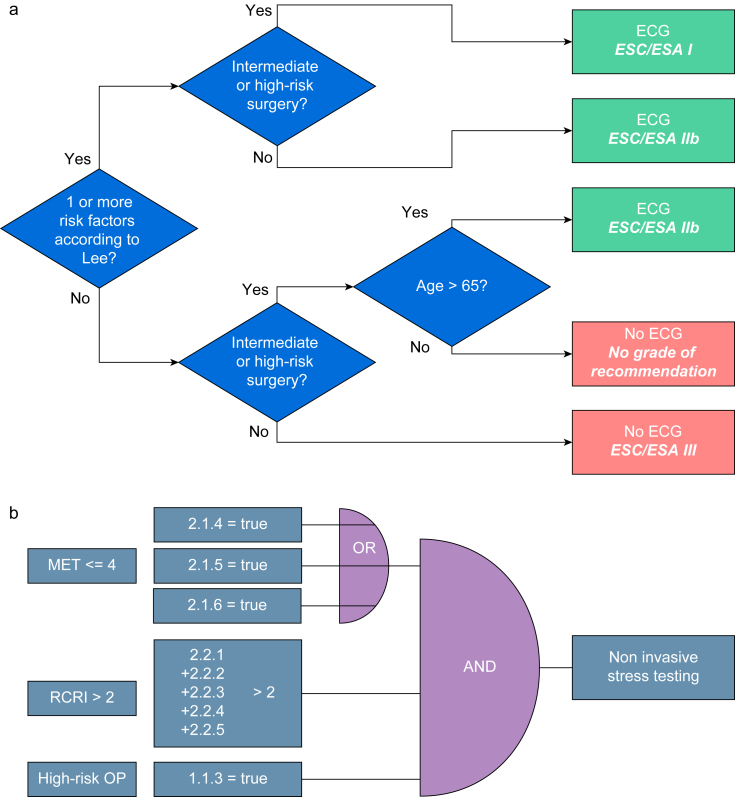
Decision rules stored as (a) dichotomous decision trees or (b) logical AND/OR combinations using ESC and ESA guideline recommendations. Examples for the translation of the guidelines into a decision support tool: (a) Flow diagram of conditions leading to the recommendation of ECG. (b) Logical AND/OR links leading to the recommendation of non-invasive cardiac stress testing. The numbers correspond to the entered observations' database identifiers (primary keys). The relevant observations, in this case, concern the MET as a measure of functional capacity represented by observations (2.1.4, 2.1.5, or 2.1.6) linked by an OR-condition and the RCRI as a score calculated from observations 2.2.1–2.2.5 indicating the presence of cardiac risk factors and high-risk surgical conditions represented by observation 1.1.3. ESA, European Society of Anaesthesiology; ESC, European Society of Cardiology; MET, metabolic equivalent of task; RCRI, revised cardiac risk index, OP, operation.

The software outputs applicable recommendations, including their grades and levels of evidence according to GRADE,^9^ if any. If a recommendation is based on multiple conditions, the software displays only the one with the highest GRADE level, whereas all other applicable conditions remain available in the database. In addition, risk scores are calculated from the information entered, such as the Preoperative Score to Predict Postoperative Mortality (POSPOM),^10^ the Charlson Comorbidity Index,^11^ the STOP-BANG score for screening of obstructive sleep apnoea,^12^ and the General Surgery Acute Kidney Risk Index to calculate the risk of perioperative renal failure.^13^ An example of the output with calculated scores and guideline recommendations is shown in Supplementary Figure S2.

Given the comprehensive availability of the software's guideline knowledge and its strict adherence, we hypothesise that the preoperative evaluation with guideline-based digital decision support is more in agreement with that of an expert than the standard approach in our centre.

In this prospective study, we analysed the results of preoperative evaluation, that is, clearance for elective surgery, in high-risk adult patients. We investigated the agreement between an anaesthetist's decision using the digital guideline support tool and the decision made by an experienced anaesthesiologist specifically trained in all applicable guidelines (the ‘expert’). The decision made by the anaesthetists on duty to clear these patients for surgery was also tested for agreement with the expert's decision (standard approach). The levels of agreement of the two clearance approaches with the expert were compared. In addition, agreement was investigated for more extensive examinations beyond medical history and physical examination for the decision on clearance for surgery.

## Methods

### Study design

After approval by the Ethics Committee of the Medical Faculty of the Technical University of Munich (TUM), Munich, Germany (Chairperson G. Schmidt, ethics committee number 38/19 S-SR from July 25, 2019) and Clinical Trials registration (ClinicalTrials.gov ID NCT04058769), this single-centre prospective agreement study was conducted from June 2021 to June 2022 at the Department of Anaesthesiology of a German university hospital (Klinikum rechts der Isar of the Technical University of Munich). This paper was written according to the Guidelines for Reporting Reliability and Agreement Studies (GRRAS).^14^

### Patients and randomisation

Patients who were undergoing elective noncardiac surgery were included in this study. Further inclusion criteria were age ≥18 yr and ASA physical status 3 or 4. A staff member who was not involved in the preoperative patient evaluation informed the patients about the study, asked them to sign the informed consent form, and organised randomisation and blinding of the assessors.

Each patient was evaluated before the operation using two approaches: the standard approach for preoperative evaluation and the digital guideline support approach. Patients were randomly assigned in a 1:1 ratio to the sequence in which the two preoperative evaluation approaches were to be performed to equally distribute carry-over effects. This means that all patients were evaluated twice: 50% of the patients were first evaluated using the standard approach and afterwards using the digital guideline support approach; 50% were evaluated the other way around.

### Intervention

The anaesthetists on duty conducted the standard preoperative evaluation according to internal standards using the in-house patient data management system, which provides the documentation of the patient's medical history, current findings, and medication history in categorical and free-text form. Because preoperative evaluation by nurses is not permitted in Germany, physicians of the Department of Anaesthesiology exclusively conducted the patients' preoperative assessment. They were trained on the guidelines for preoperative assessment as part of the weekly 45-min continuing medical education programme. Routinely, an experienced senior physician is always available in the pre-anaesthesia outpatient clinic to supervise the physicians undertaking assessments, 80–90% of whom are still in training and have not yet completed their specialist qualification. The anaesthetists performing the standard evaluation were aware of neither the study nor their patient's participation. The standard evaluation was binding as long as no violation of a GRADE 1 recommendation was identified in the subsequent expert assessments.

The preoperative evaluation using digital guideline support was performed by an anaesthetist trained in the technique, who was blinded to the results of the standard evaluation if it had been performed beforehand. This anaesthetist performed history-taking and clinical examinations exactly as suggested by the software and had to adhere to all recommendations of the guidelines.

After completing their evaluation, the anaesthetists of both evaluation approaches decided whether patients could be cleared for surgery according to the rules of the respective approach. In case of non-clearance, they ordered additional examinations. The results of both evaluation approaches were transferred to a standardised spreadsheet to be assessed by an expert blinded to the approaches.

The expert was a specialist in anaesthesiology who had thoroughly studied all guidelines used and was also allowed to look them up when needed. The results of both preoperative evaluation approaches were assessed by the expert based on the documented information on the spreadsheet and all original findings in the hospital information system to decide whether clearance for surgery should have been given according to the applicable guidelines or whether examinations were required beforehand. The expert did not visit any patient. If a GRADE 1 recommendation was violated, the expert was unblinded to allow correction.

### Outcomes

The primary outcome was the decision to clear high-risk patients for surgery, using either the digital guideline support approach or the standard approach, with the decisions of an experienced anaesthetist. A digital guideline-supported decision to clear for surgery was classified as ‘correct’ if it agreed with the expert's decision. Accordingly, there were two modes of agreement: agreement to clear and agreement not to clear for surgery. Disagreements included ‘not followed’ recommendations or clearances when the expert had not done so and ‘wrongly rejected’ conditions when clearance for the operation was not given even though the expert had given it. Analogously, agreement or disagreement was decided for the standard approach. The agreement levels of both approaches were compared with the expert's decision.

Secondary outcomes were the agreement in more extensive preoperative examinations beyond physical examination, patient and drug history, such as ECG, echocardiography, non-invasive cardiac stress tests, pulmonary function tests, carotid Doppler, or various laboratory tests, which were requested at least once by the anaesthetist, the expert, or the digital guideline support tool.

Exploratory outcomes included the time needed for completing the preoperative evaluation, several postoperative complications, and the discharge dispositions.

### Sample size consideration

For agreement studies, 200 observations were required to keep the confidence interval around the limits of agreement <0.25 standard deviations of the measurement differences.^15^ We included 204 patients, assuming a 2% loss to follow-up.

### Statistical analysis

Statistical analyses were conducted using R version 4.2.2 (R Foundation for Statistical Computing, Vienna, Austria). Continuous variables are presented as median with interquartile range or mean with standard deviations, and categorical variables as absolute numbers and frequencies.

Cohen's kappa coefficients were calculated as a measure of interrater reliability for the agreement between the expert and the digital guideline support approach and for the agreement between the expert and the standard approach in deciding whether to clear for surgery. For Cohen's kappa, the Landis and Koch reference values were used as a standard for evaluation.^16^ The coefficients of both approaches were compared using the z-test. Because of their skewed probability distribution, Gwet's first-order agreement coefficient (Gwet's AC1) was calculated for agreement analyses in the request for more extensive examinations.^17^ In analogy to the Landis and Koch reference values, coefficients of 1–0.8 indicate very good agreement, 0.8–0.6 good agreement, 0.6–0.4 moderate agreement, 0.2–0.4 fair agreement, and <0.2 poor agreement.^18^

Frequencies were compared between approaches using proportion tests, Fisher's exact test, or χ^2^ test, and continuous variables using Students *t*-test or Wilcoxon rank sum test, as indicated. A *P*-value of 5% was defined as significant.

## Results

A total of 204 patients for elective surgery at high perioperative risk were included, none of whom were lost during the study. Patient characteristics are given in Table 1.

**Table 1:** Patient characteristics. Values are medians (interquartile range) or numbers of patients (%). Preoperative score to predict postoperative mortality (POSPOM).^10^ Charlson Comorbidity Index.^11^ The surgical risk (30-day risk of cardiovascular death and myocardial infarction) was determined according to the 2014 European Society of Cardiology guidelines.^4^

Characteristic	Value
Biometrics	
Age (yr)	73 (64–79)
Female sex	99 (48.5)
Body mass index (kg m^−2^)	25 (22–29)
ASA physical status	
3	200 (98.0)
4	4 (2.0)
Surgical risk	
Low (<1%)	21 (10.3)
Intermediate (1–5%)	131 (64.2)
High (>5%)	52 (25.5)
POSPOM	29 (26–32)
Charlson Comorbidity Index	
0	46 (22.5)
1–2	111 (54.4)
3–4	22 (10.8)
≥5	3 (1.5)
Unknown	22 (10.8)

### Clearance for surgery

In the digital guideline support approach, 37 (18.1%) patients were cleared for surgery without further investigations; in the standard approach, 151 (74.0%) were cleared; and with the expert, 97 (47.5%) were cleared. Both agreements, the one between the clearance decisions using digital guideline support and those of the expert (66.7%) and the one between the standard clearance decisions and those of the expert (67.6%), were fair, with no significant difference in the coefficients (Cohen's kappa 0.37 [interquartile range 0.26–0.48] *vs* 0.31 [0.21–0.42], *P*=0.6; Table 2). The correct clearance decisions using digital guideline support mainly consisted of patients with correctly requested additional examinations in 50.5%, whereas correct clearance decisions using the standard approach mainly consisted of patients correctly cleared for surgery in 44.6% (Table 2). The wrong decisions using digital guideline support were 31.4%, based on unnecessarily requested additional examinations, whereas the wrong decisions of the standard approach were 29.4% based on recommendations not followed. Characteristics of patients cleared by the standard approach but not by the digital guideline support approach are reported in Supplementary Table S4.

**Table 2:** Agreement between clearance decisions for elective surgery in 204 patients at high perioperative risk using digital guideline support and an expert's decision compared with the agreement of standard clearance decisions with an expert's decision. The values are the number of patients (%) whose preoperative evaluation with the respective approach was judged correct or incorrect by the expert. Correctly evaluated patients were either correctly cleared or correctly rejected (non-clearance) for surgery. Patients with incorrect evaluations were either incorrectly cleared for surgery because examinations were not performed that the expert considered necessary (not followed recommendations) or incorrectly not cleared for surgery because unnecessary examinations were requested (incorrectly rejected).

	Digital guideline support	Standard	*P*-value
Cohen's kappa	0.37 (0.26–0.48)	0.31 (0.21–0.42)	0.6
Correctly cleared for surgery	33 (16.2)	91 (44.6)	<0.001
Correctly rejected	103 (50.5)	47 (23.0)	<0.001
Not followed recommendations	4 (2.0)	60 (29.4)	<0.001
Incorrectly rejected	64 (31.4)	6 (2.9)	<0.001

### Apparative and laboratory examinations

**Fig 2:**
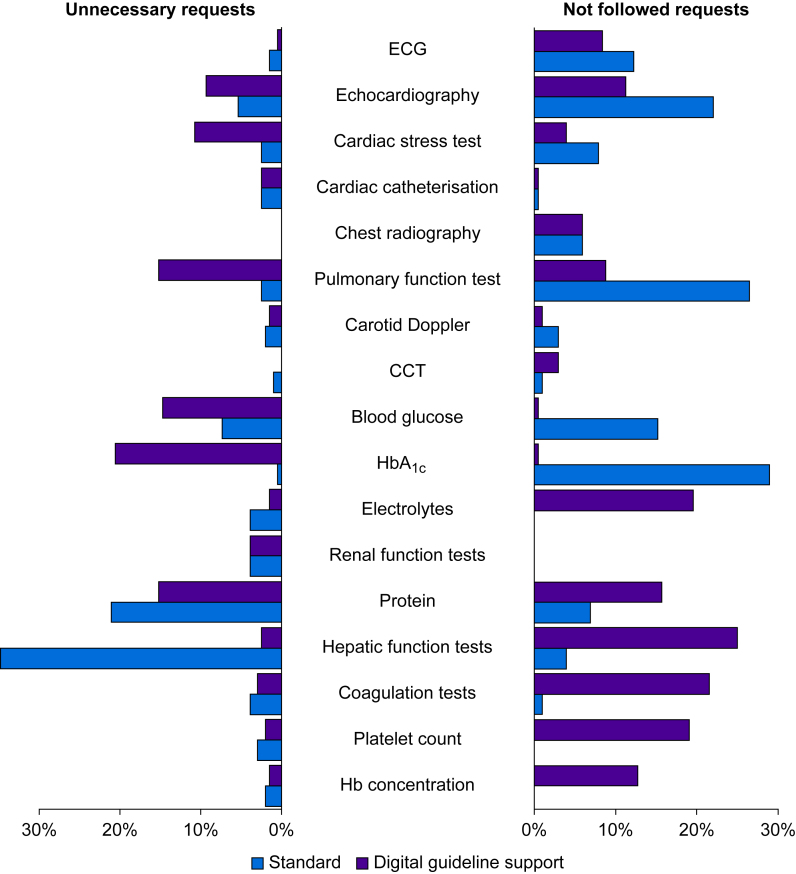
Frequencies and types of incorrect requests for more extensive examinations following the preoperative evaluation using the digital guideline-supported approach and the standard approach. The bars on the right side show how frequent (in relation to all patients) more extensive examinations were not performed that the expert considered necessary (‘Not followed requests’). The bars on the left side show how frequently more extensive examinations were requested that the expert considered unnecessary (‘Unnecessary requests’). The purple bars show the incorrect requests of the approach supported by the digital guideline support, and the blue bars show those of the standard approach. CCT, cranial computed tomography; ECG, electrocardiography; Hb, haemoglobin; HbA_1c_, glycated haemoglobin.

**Fig 3:**
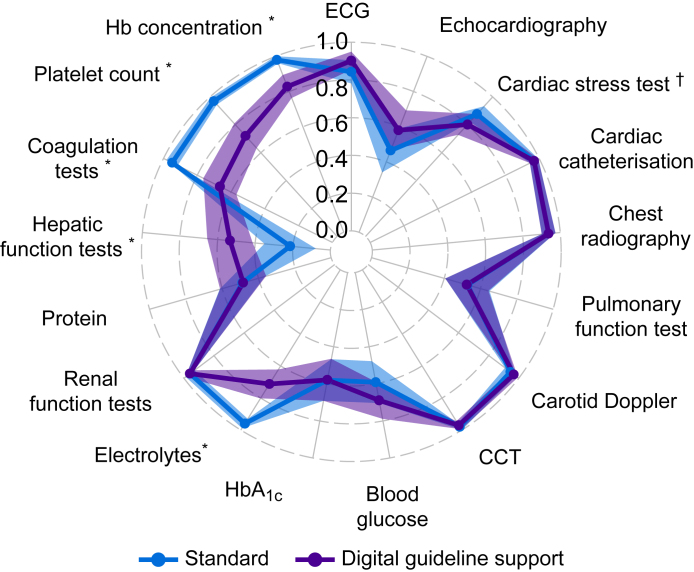
Agreement between the requests of more extensive examinations by the digital guideline-based approach and the standard approach and those of the expert. The radar plot shows Gwet's AC1 and their 95% confidence intervals for the agreement between digital guideline-supported requests (purple) and the standard approach (blue), each with the expert. Coefficients are given for every more extensive preoperative examination beyond physical examination, patient, and drug history. The plot displays values from 0 to 1 only because negative values indicating less agreement than by chance did not appear. Coefficients of 1.0–0.8 indicate very good agreement, 0.8–0.6 good agreement, 0.6–0.4 moderate agreement, 0.2–0.4 fair agreement, and <0.2 poor agreement.^17^ ∗*P*<0.05 for comparison of the approaches. ^†^Cardiac stress test includes stress echocardiography and myocardial scintigraphy. CCT, cranial computed tomography; ECG, electrocardiography; Hb, haemoglobin; HbA_1c_, glycated haemoglobin.

Using the digital guideline support approach, all 1193 ESAIC guideline recommendations for more extensive examinations were adhered to (eight of GRADE 1A, 13 of GRADE 1B, 191 of GRADE 1C, 362 of GRADE 2A, 378 of GRADE 2B, and 241 of GRADE 2C). Based on the expert's assessment, the need for laboratory values before major abdominal surgery in the context of expected major bleeding and volume shifts was underestimated by the digital guideline support approach, including electrolytes, protein, hepatic function tests, coagulation tests, platelet count, and haemoglobin concentration in ∼20% of patients. To a lesser extent (<10%), the need for ECG, echocardiography, cardiac stress tests, chest X-ray, and pulmonary function tests was also underestimated. In other patients, echocardiography, cardiac stress tests, and pulmonary function tests were requested unnecessarily (∼10%). The request for blood glucose, HbA_1c_ (glycated haemoglobin), and protein values were not confirmed by the expert in 15–20% (Fig. 2). Gwet's AC1 showed very good agreement in requesting more extensive examinations to assess the cardiovascular risk, good agreement in assessing bleeding risk, and moderate agreement in assessing pulmonary and metabolic risk (Fig. 3; numbers are given in Supplementary Table S1).

Using the standard approach, 830 (70%) guideline recommendations for more extensive examinations were adhered to, but not six GRADE 1A, five GRADE 1B, and 30 GRADE 1C recommendations, accounting for 37 patients. The expert intervened in five patients with violations of GRADE 1A or 1B recommendations; ECGs were to be performed in two patients and echocardiograms in three, all of which revealed no critical findings.

Based on the expert's assessment, necessary requests for more extensive examinations in obese patients (i.e. pulmonary function tests [26%], HbA_1c_ [29%], and blood glucose values [15%]), and in others, the request for echocardiography (22%) and ECG (12%) were not followed using the standard approach. Unnecessary protein and hepatic function tests were ordered in 21% and over 30% of patients, respectively (Fig. 2). Gwet's AC1 showed very good agreement in requesting more extensive examinations to assess bleeding risk, renal function, and cardiovascular risk, except for moderate agreement for echocardiography. Moderate agreement was also found for further clarification of pulmonary function and diabetes mellitus. Fair agreement was found for the assessment of hepatic function (Fig. 3; numbers are given in Supplementary Table S1).

In 140 of 204 patients, 212 GRADE 1A–C of 1193 ESAIC recommendations (17.8%) were effective. Using the digital guideline support approach, all these recommendations were followed, whereas the standard approach covered only 81%. The expert considered six GRADE 1C recommendations (3%) not applicable. Gwet's AC1 showed very good agreement (expert *vs*. digital guideline support: 0.95; expert *vs*. standard approach: 0.84).

### Duration of approaches

The standard approach required an average of 25.2 (sd 10.8) min, whereas the digital guideline support approach required an average of 37.3 (6.9) min (*P*<0.001).

### Clinical outcomes

After completing all more extensive examinations indicated by the standard approach and, if necessary, the expert correction and suggested preoperative treatment, patients underwent surgery, with two exceptions. One patient opted for conservative treatment after preoperative assessment, and another died before surgery. Patients stayed in the hospital for 13 (8–21) days; 64 of them (31.4%) needed intensive care for at least 1 (1–3) day, and 42 (20.6%) required revision surgery. After the treatment, 122 (59.8%) patients were discharged home, 77 (37.7%) needed further treatment in any hospital or rehabilitation facility, and five (2.5%) died during hospital stay (for causes of death, see Supplementary Table S2).

The exploratory comparison of patient trajectories in terms of clearance decisions between the two evaluation approaches and those of the expert revealed that two patients died after being cleared by the expert and the standard approach, whereas none died after clearance by the digital guideline support approach (Table 3 and Supplementary Tables S3 and S4).

**Table 3:** Outcome of patients cleared for surgery without additional examinations evaluated using the digital guideline support approach compared with patients additionally cleared using the standard approach (exploratory analysis). Values are numbers of patients (%) or medians (interquartile range). For details, see Supplementary Tables S3 and S4. Statistical power was <0.4 in all analysed outcomes. ∗Two of the 37 patients were not cleared with the standard approach. ^†^The 35 patients also cleared with the digital guideline support approach are not listed here.

Outcome	Digital guideline support (*n=*37)∗	Standard (*n=*116)^†^	*P*-value
Required surgical re-intervention	4 (10.8)	26 (22.4)	0.1
ICU admission	9 (24.3)	36 (31.0)	0.4
Need for artificial ventilation	2 (5.4)	5 (4.3)	0.7
Length of ICU stay (days)	1 (1–1)	1 (1–2)	0.4
Hospital length of stay (days)	11 (6–18)	13 (8–21)	0.2
Discharged home	21 (56.8)	76 (65.5)	0.3
Follow-up treatment/rehabilitation	16 (43.2)	53 (45.7)	0.2
In-hospital mortality	0 (0.0)	2 (1.7)	0.9

## Discussion

In this prospective study, we were unable to demonstrate that a guideline-based digital-supported decision preoperative assessment was more in agreement with that of an expert than a standard approach. Both agreement levels concerning the decision to clear high-risk patients for elective surgery were only fair. However, the background of the two non-agreements differed considerably. The lack of agreement with the expert was based on 31.4% of over-cautious non-clearance decisions using the digital guideline support approach and 29.4% of recommendations that were not followed using the standard approach. Furthermore, disagreement could not be attributed to any specific examination or disease complex but was attributed to the sum of the various examinations.

The key question of a preoperative evaluation is whether a patient can be cleared for surgery or if any more extensive examination or intervention is needed to gain further information, improve clinical status, or optimise perioperative care. In the case of elective surgery, which by definition can be postponed, a surgery delay is justified. With rigorous adherence to the guidelines realised by the digital guideline support tool, only 18.1% of the investigated high-risk patients were cleared for surgery without additional examinations. Such a strict approach would hardly have been accepted in our patient cohort because many of the interventions were scheduled as elective but could not be postponed for any length of time. In addition, the inconvenience for the patients resulting from ordering additional examinations and postponing the surgery must also be considered.

Guideline authors themselves point out that the recommendations must always be adapted to the context of an individual patient and the organisational circumstances of the clinical environment.^19^ Experienced anaesthetists relativise indications for additional examinations (e.g. in palliative patients), prioritise recommendations in multimorbid patients by interdisciplinary consensus (e.g. insertion of a cardiac stent before or after cancer surgery), or include knowledge about the surgical risks (e.g. bleeding and transfusion likelihood).^4^^,^^20^ By doing so, the expert would have cleared 47.5% of the high-risk patients in this study for surgery without additional examinations. Possibly, the digital system identified risks that the expert failed to recognise, knowingly accepted, or considered irrelevant to the outcome.

As the guidelines are not legally binding, an anaesthetist's deviation from the recommended procedure has just as little consequence as a deviation of in-house standard operating procedures from the guidelines. Moreover, the value of strict guideline recommendations is increasingly being questioned.^21^ For example, in a recent study, the more stringent ESC recommendation of 2022 for the determination of cardiac biomarkers was ignored without this having any effect on the outcome.^22^

If two simultaneously applicable guideline recommendations contradict each other, non compliance with at least one is inevitable. An example is the recommendation for preoperative echocardiography before noncardiac surgery. The German guidelines recommend preoperative echocardiography only for new onset dyspnoea of unknown cause or worsening of known heart failure. In contrast, the ESC guidelines recommend current transthoracic echocardiography in patients with heart failure before any medium- or high-risk surgery or, alternatively, a determination of natriuretic peptides (class 1A). This also inevitably results in a high probability of a lack of agreement between the evaluation approaches analysed.

Although outcomes are critically important, they are frequently not sensitive enough to capture the nuances of each decision, especially those that arise during preoperative assessment. This agreement study was not designed, nor was the sample size estimated, to account for potential differences in outcomes depending on the preoperative evaluation approach. However, as unfavourable outcomes frequently occurred in our study cohort of ASA physical status 3–4 patients, our data can be used to estimate the sample size of a future randomised controlled trial, testing whether adherence to guidelines using this software can improve outcomes.

The exploratory analysis of the outcomes of patients who were cleared for surgery according to the standard approach, but not according to the digital guideline support approach, tended to have a more complicated postoperative course. None of the five patients dying in the hospital would have been cleared for surgery at the first anaesthesia consultation with digital guideline support, whereas both the expert and the anaesthetists using the standard assessment had cleared two of them: one whose surgery was for symptom control under comfort care and a second one whose infection unexpectedly exacerbated into sepsis after being cleared for surgery. It is important to note that these results are only exploratory and, at best, serve to generate hypotheses. It is certainly not permitted to conclude that adherence to the guideline recommendations could have led to a better outcome.

If the generally good to very good agreement of both the digital guideline support and standard approaches with the expert in individual examinations was moderate or even fair, it was not because of a generally more liberal or restrictive approach. Some clinical conditions are not covered by current guidelines, such as more extensive examinations regarding the planned surgery (e.g. coagulation tests expecting major bleeding and glucose levels before pancreatic surgery), further examinations undergoing preoperative neoadjuvant chemotherapy (e.g. blood cell counts, renal function tests, and hepatic function tests), chronic pain therapy with nonsteroidal anti-inflammatory agents (e.g. renal function tests and hepatic function tests), or drug abuse (e.g. hepatic and renal function tests). However, our results suggest that knowledge of guidelines and their presentation by digital guideline support can save a considerable number of unnecessary preliminary examinations.

The preoperative examination took longer with digital guideline support than with the standard approach, as a detailed history and documentation are mandatory. In addition, the lack of interfaces between the nonclinical software package and the hospital information system forced the anaesthetist to manually enter laboratory values and examination results. In contrast, an average duration of less than 30 min for the examination of a high-risk patient may be too short and may have contributed to necessary examinations being overlooked. It may also reflect the high level of routine with the standard approach. A definitive analysis of the costs of both procedures can only be performed once such a software tool is integrated into the hospital information system with all the necessary interfaces and the staff has developed a routine for using it.

Finally, this study highlights the conceptual shortcomings of the guidelines in their current design. In this cohort of high-risk surgical patients, an average of six ESAIC recommendations were applied to each patient. However, only one of 10 patients had a GRADE 1A or GRADE 1B recommendation (i.e. a strong recommendation with high-quality or moderate-quality evidence). Regarding additional examinations, 82.2% of the corresponding recommendations were weak, and a further 16.0%, although strong, were based on low-quality evidence. Adherence to GRADE 1A-C recommendations was remarkably higher even using the standard approach and in very good agreement with the expert. Using this cohort of high-risk patients as an example, two fundamental considerations arise for the digitalisation of guidelines for decision support systems, but even more so for the physicians who use them: (1) how can there be strong recommendations without scientific evidence at all (GRADE 1C) and (2) how to deal with weak recommendations (GRADE 2A-C). At least, they are not binding.

### Strengths and limitations

We present a single-centre study conducted at a large German university hospital with a high proportion of residents who, however, always have an experienced senior physician available to answer their questions. Therefore, our results regarding guideline adherence may only be partially transferable to other hospitals.^23^ However, the usefulness of a digital decision support tool applies independently. Furthermore, we have digitalised the guideline recommendations through expert consensus because unclear wording with room for interpretation and the limited availability of machine-readable guidelines make it difficult to establish clear rules.^24^

In this study, we recruited ASA physical status 3 and 4 patients, which limits generalisability. However, ASA physical status 3 and 4 patients were particularly suitable for the study objective of investigating the agreement of different preoperative evaluation procedures, as they trigger the majority of guideline recommendations owing to their pre-existing conditions.

It must also be noted that the expert was only one person. It cannot be ruled out that other experienced anaesthetists would have deviated from this expert's decisions. In particular, a high level of agreement between the standard approach and the expert could be because of affiliation with the same clinic. However, a guideline expert may be in favour of strict adherence. In fact, the expert's decisions were between the more generous clearance of the standard approach and the restrictive one of digital guideline support. Nevertheless, the risk of limited generalisability is associated with the restriction to one expert.

Given the significantly different ways of taking the medical history between the highly structured, guideline-based approach and the standard approach, the patients' statements were most likely also different. Missing structure also allows room for interpretation of even identical statements. However, we cannot comment on the extent of the differences because no witnesses were present because of the blinding of the anaesthetists on duty. Importantly, a structured patient history is part of guideline adherence and quality assurance.

The present investigation is a proof-of-concept study using software on the level of a demonstrator. For routine clinical use, the software would probably have to be certified as a medical device. The potential of digital decision support to improve the quality of medical care depends on further factors, for example, usability and user satisfaction, design and application features such as Named Entity Recognition or even Natural Language Processing.^25^^,^^26^ Furthermore, it will be of interest to evaluate the software at other than academic centres. These issues still need to be addressed in future studies.

### Conclusions

In summary, digital guideline support is not flexible enough to be sufficiently adaptable to the individual patient's clinical context and the clinical setting's organisational realities. Nevertheless, the digitalisation of guidelines can make their knowledge available in the context of individual patients, thus training anaesthetists in using guidelines.^7^^,^^27^ However, vaguely written recommendations cannot be translated into digital rules. Weak recommendations or low-quality evidence are not suitable for decision support. At best, they can serve as a nonbinding source of information; otherwise, they contribute to overly restrictive clearance for surgery.

## Authors’ contributions

Designed the study: BJ, SMK, MB

Performed data acquisition: JW, DIA

Conducted the analysis: BU, AHP, SMK, JW

Performed data interpretation: MB, BJ, SMK, GS

Drafted the manuscript: JW, SMK

Wrote the second draft: MB

All authors critically revised the manuscript for important intellectual content and approved the final version to be published: all authors

Agree to be accountable for all aspects of the work in ensuring that questions related to the accuracy or integrity of any part of the work are appropriately investigated and resolved: all authors

## Acknowledgements

The authors are indebted to Koen Coekaerts and Javier Ávila Solbes, Health Information Management GmbH (HIM™) for the development and special adaptation of the software for study purposes.

## Declarations of interest

SMK and BJ received grants from Löwenstein Medical Innovation™ (Berlin, Germany). MB received research support from MSD™ (Haar, Germany), fees for consultancy or lectures from GE Healthcare™ (Helsinki, Finland), Grünenthal™ (Aachen, Germany), and Senzime™ (Landshut, Germany). All other authors declare that they have no competing interests. No author holds shares or receives fees from HIM™ or has any other financial interests.

## Funding

The project was funded by the Central Innovation Program for small and medium-sized enterprises of the German 10.13039/501100006360Federal Ministry for Economic Affairs and Energy (ZF4544701TS8) as a joint project between TUM and HIM™ (Health Information Management GmbH, Bad Homburg, Germany) acting as cooperation partners. HIM™ was solely responsible for the software programming and had no influence on the study design, nor was it involved in the data analysis. Neither HIM™ nor the Federal Ministry influenced the study design, the analysis, and the interpretation of the data and the report's writing.

## Data availability statement

Because of legal requirements, we are not allowed to store data, although they are de-identified, in a publicly accessible repository. To gain access, proposals should be directed to the corresponding author. Requestors will need to sign a data access agreement.
