# Supplementary material for: Implementation of a software-based decision support tool for guideline-appropriate preoperative evaluation: a prospective agreement study
Source: Br J Anaesth. 2024 Jul 5;133(3):519–29. doi: 10.1016/j.bja.2024.06.001 (PMC11347788; doi:10.1016/j.bja.2024.06.001)
Supplement: Multimedia component 1 [file mmc1.docx]

# Supplementary Material

|  |
| --- |
| **Fig. S1** User interface providing digital-guideline-support (translated from German).  Avoidance of free text entries and hierarchical entry structure, mandatory fields are highlighted in orange. |

|  |
| --- |
| **Fig. S2** Summary of evaluation results through the new tool. Overview of findings, scores and guideline recommendations (translated from German) |

| \| **Table S1**: Cross-tables for agreement between standard approach and expert as well as digital-guideline-supported approach and expert.  The number of results matching the expert is the sum of the diagonals from top left to bottom right in each cross-table. Confidence Intervals are given in square brackets. Gwet's AC1 indicates the agreement between expert and the respective assessment mode for each examination.  ECG, electrocardiography; CCT, cranial computer tomography; Hb, haemoglobin; HbA_1c_, glycated haemoglobin. \| \| \| \| \| \| \| \| \| \| \| \| --- \| --- \| --- \| --- \| --- \| --- \| --- \| --- \| --- \| --- \| --- \| \| **Parameter** \| **Standard vs. expert** \| \| \| \| \| **Digital-guideline-support vs. expert** \| \| \| \| \| \|  \|  \| **Expert** \| \| \| **Gwet's AC1** \|  \| **Expert** \| \| \| **Gwet's AC1** \| \|  \| **standard** \|  \| yes \| no \|  \| **digital-guideline-support** \|  \| yes \| no \|  \| \| **ECG** \| yes \| 176 \| 3 \| **0.84 [0.78 to 0.90]** \| yes \| 184 \| 1 \| **0.90 [0.85 to 0.95]** \| \| no \| 25 \| 0 \| no \| 17 \| 2 \| \|  \|  \| yes \| no \|  \|  \| yes \| no \|  \| \| **Echocardiography** \| yes \| 52 \| 11 \| **0.46 [0.34 to 0.59]** \| yes \| 74 \| 19 \| **0.58 [0.47 to 0.69]** \| \| no \| 46 \| 95 \| no \| 24 \| 87 \| \|  \|  \| yes \| no \|  \|  \| yes \| no \|  \| \| **Pulmonary function test** \| yes \| 24 \| 5 \| **0.53 [0.41 to 0.65]** \| yes \| 59 \| 31 \| **0.52 [0.41 to 0.64]** \| \| no \| 54 \| 121 \| no \| 19 \| 95 \| \|  \|  \| yes \| no \|  \|  \| yes \| no \|  \| \| **Myocardial stress test** \| yes \| 8 \| 5 \| **0.88 [0.82 to 0.93]** \| yes \| 16 \| 22 \| **0.80 [0.73 to 0.88]** \| \| no \| 16 \| 175 \| no \| 8 \| 158 \| \|  \|  \| yes \| no \|  \|  \| yes \| no \|  \| \| **Cardiac catheterization** \| yes \| 1 \| 5 \| **0.97 [0.95 to 0.99]** \| yes \| 1 \| 5 \| **0.97 [0.95 to 0.99]** \| \| no \| 1 \| 197 \| no \| 1 \| 197 \| \|  \|  \| yes \| no \|  \|  \| yes \| no \|  \| \| **Carotid doppler** \| yes \| 8 \| 4 \| **0.94 [0.91 to 0.98]** \| yes \| 11 \| 3 \| **0.97 [0.94 to 0.99]** \| \| no \| 6 \| 186 \| no \| 3 \| 187 \| \|  \|  \| yes \| no \|  \|  \| yes \| no \|  \| \| **Chest X-ray** \| yes \| 0 \| 0 \| **0.94 [0.90 to 0.97]** \| yes \| 0 \| 0 \| **0.94 [0.90 to 0.97]** \| \| no \| 12 \| 192 \| no \| 12 \| 192 \| \|  \|  \| yes \| no \|  \|  \| yes \| no \|  \| \| **Blood glucose** \| yes \| 46 \| 15 \| **0.59 [0.48 to 0.71]** \| yes \| 76 \| 31 \| **0.69 [0.59 to 0.79]** \| \| no \| 31 \| 112 \| no \| 1 \| 96 \| \|  \|  \| yes \| no \|  \|  \| yes \| no \|  \| \| **HbA1c** \| yes \| 5 \| 1 \| **0.58 [0.47 to 0.69]** \| yes \| 64 \| 43 \| **0.58 [0.47 to 0.69]** \| \| no \| 60 \| 138 \| no \| 1 \| 96 \| \|  \|  \| yes \| no \|  \|  \| yes \| no \|  \| \| **Hepatic function tests** \| yes \| 72 \| 73 \| **0.21 [0.08 to 0.35]** \| yes \| 28 \| 5 \| **0.53 [0.41 to 0.65]** \| \| no \| 8 \| 51 \| no \| 52 \| 119 \| \|  \|  \| yes \| no \|  \|  \| yes \| no \|  \| \| **Electrolytes** \| yes \| 196 \| 8 \| **0.96 [0.93 to 0.99]** \| yes \| 154 \| 3 \| **0.71 [0.62 to 0.80]** \| \| no \| 0 \| 0 \| no \| 42 \| 5 \| \|  \|  \| yes \| no \|  \|  \| yes \| no \|  \| \| **Renal function tests** \| yes \| 196 \| 8 \| **0.96 [0.93 to 0.99]** \| yes \| 196 \| 8 \| **0.96 [0.93 to 0.99]** \| \| no \| 0 \| 0 \| no \| 0 \| 0 \| \|  \|  \| yes \| no \|  \|  \| yes \| no \|  \| \| **protein** \| yes \| 39 \| 44 \| **0.49 [0.36 to 0.61]** \| yes \| 20 \| 32 \| **0.48 [0.36 to 0.61]** \| \| no \| 14 \| 107 \| no \| 33 \| 119 \| \|  \|  \| yes \| no \|  \|  \| yes \| no \|  \| \| **Hb-concentration** \| yes \| 200 \| 4 \| **0.98 [0.96 to 1.00]** \| yes \| 173 \| 3 \| **0.83 [0.76 to 0.89]** \| \| no \| 0 \| 0 \| no \| 27 \| 1 \| \|  \|  \| yes \| no \|  \|  \| yes \| no \|  \| \| **Platelets** \| yes \| 198 \| 6 \| **0.97 [0.95 to 0.99]** \| yes \| 157 \| 4 \| **0.72 [0.63 to 0.81]** \| \| no \| 0 \| 0 \| no \| 41 \| 2 \| \|  \|  \| yes \| no \|  \|  \| yes \| no \|  \| \| **Coagulation tests** \| yes \| 194 \| 8 \| **0.95 [0.92 to 0.98]** \| yes \| 150 \| 6 \| **0.67 [0.57 to 0.76]** \| \| no \| 2 \| 0 \| no \| 46 \| 2 \| |
| --- | --- | --- | --- | --- | --- | --- | --- | --- | --- | --- | --- | --- | --- | --- | --- | --- | --- | --- | --- | --- | --- | --- | --- | --- | --- | --- | --- | --- | --- | --- | --- | --- | --- | --- | --- | --- | --- | --- | --- | --- | --- | --- | --- | --- | --- | --- | --- | --- | --- | --- | --- | --- | --- | --- | --- | --- | --- | --- | --- | --- | --- | --- | --- | --- | --- | --- | --- | --- | --- | --- | --- | --- | --- | --- | --- | --- | --- | --- | --- | --- | --- | --- | --- | --- | --- | --- | --- | --- | --- | --- | --- | --- | --- | --- | --- | --- | --- | --- | --- | --- | --- | --- | --- | --- | --- | --- | --- | --- | --- | --- | --- | --- | --- | --- | --- | --- | --- | --- | --- | --- | --- | --- | --- | --- | --- | --- | --- | --- | --- | --- | --- | --- | --- | --- | --- | --- | --- | --- | --- | --- | --- | --- | --- | --- | --- | --- | --- | --- | --- | --- | --- | --- | --- | --- | --- | --- | --- | --- | --- | --- | --- | --- | --- | --- | --- | --- | --- | --- | --- | --- | --- | --- | --- | --- | --- | --- | --- | --- | --- | --- | --- | --- | --- | --- | --- | --- | --- | --- | --- | --- | --- | --- | --- | --- | --- | --- | --- | --- | --- | --- | --- | --- | --- | --- | --- | --- | --- | --- | --- | --- | --- | --- | --- | --- | --- | --- | --- | --- | --- | --- | --- | --- | --- | --- | --- | --- | --- | --- | --- | --- | --- | --- | --- | --- | --- | --- | --- | --- | --- | --- | --- | --- | --- | --- | --- | --- | --- | --- | --- | --- | --- | --- | --- | --- | --- | --- | --- | --- | --- | --- | --- | --- | --- | --- | --- | --- | --- | --- | --- | --- | --- | --- | --- | --- | --- | --- | --- | --- | --- | --- | --- | --- | --- | --- | --- | --- | --- | --- | --- | --- | --- | --- | --- | --- | --- | --- | --- | --- | --- | --- | --- | --- | --- | --- | --- | --- | --- | --- | --- | --- | --- | --- | --- | --- | --- | --- | --- | --- | --- | --- | --- | --- | --- | --- | --- | --- | --- | --- | --- | --- | --- | --- | --- | --- | --- | --- | --- | --- | --- | --- | --- | --- | --- | --- | --- | --- | --- | --- | --- | --- | --- | --- | --- | --- | --- | --- | --- | --- | --- | --- | --- | --- | --- | --- | --- | --- | --- | --- | --- | --- | --- | --- | --- | --- | --- | --- | --- | --- | --- | --- | --- | --- | --- | --- | --- | --- | --- | --- | --- | --- | --- | --- | --- | --- | --- | --- | --- | --- | --- | --- | --- | --- | --- | --- | --- | --- | --- | --- | --- | --- | --- | --- | --- | --- | --- | --- | --- | --- | --- |

| **Table S2.** Causes of death in the five patients who died during the hospital stay | | | |
| --- | --- | --- | --- |
| Type of surgery and cause of death | Clearance by Expert | Clearance by digital-guideline-support | Clearance by Standard |
| Advanced tumour disease, diagnostic panendoscopy, patient dies before transfer to palliative care unit, probably exsiccosis due to advanced ENT tumour and restricted food intake | no | no | no |
| Surgery: hip prosthesis, death due to intraoperative pulmonary embolism | no | no | no |
| Intraoperative need for cardiopulmonary resuscitation during hip prosthesis explantation (haemorrhagic shock). In the further course, sepsis and multiple organ failure | no | no | no |
| Staphylococcal infection, patient was released for focal sanitation, deterioration to sepsis before surgery | yes | no | yes |
| Hospitalized due to cervical spine stabilisation for pathological fracture, palliative DJ system, death due to underlying disease on palliative care ward | yes | no | yes |

| **Table S3.** Patient outcome related to clearance for surgery without more extensive examinations requested at the standard, the digital guideline-supported, or the expert’s evaluation (exploratory analysis).  Values are numbers of patients (%) or medians [IQR]. Since all patients underwent surgery, the assignment ‘cleared’ or ‘not cleared’ refers to whether or not further examinations were requested in the respective approach during the first consultation.  Frequencies were analysed by means of Fisher’s exact test or χ^2^-test, continuous variables were analysed by Wilcoxon rank sum test, p$\leq$0.05 was considered statistically significant. | | | | | | | | | | | | | | | |
| --- | --- | --- | --- | --- | --- | --- | --- | --- | --- | --- | --- | --- | --- | --- | --- |
| **Evaluation** | **standard** | | | | | **digital guideline-support** | | | | | **expert** | | | | |
| **Outcome** | **Cleared *n*=151** | | **Not Cleared *n*=53** | | ***P*-value** | **Cleared *n*=37** | | **Not cleared *n*= 167** | | ***P*-value** | **Cleared *n*= 97** | | **Not cleared *n*= 107** | | ***P*-value** |
| Revision surgery | 30 | (19.9) | 12 | (22.6) | 0.7 | 4 | (10.8) | 38 | (22.8) | 0.1 | 16 | (16.5) | 26 | (24.3) | 0.2 |
| Intensive care unit (ICU) | 45 | (29.8) | 19 | (35.8) | 0.4 | 9 | (24.3) | 55 | (32.9) | 0.3 | 26 | (26.8) | 38 | (35.5) | 0.2 |
| Length of ICU stay [days] | 1 | [1 -2] | 1 | [1 - 5] | >0.9 | 1 | [1 - 1] | 1 | [1 - 3] | 0.5 | 1 | [1 - 2] | 1 | [1 - 4] | 0.5 |
| Need for artificial ventilation | 7 | (4.6) | 6 | (11.3) | 0.1 | 2 | (5.4) | 11 | (6.6) | >0.9 | 3 | (3.1) | 10 | (9.3) | 0.068 |
| Hospital length of stay [days] | 13 | [7 - 20] | 15 | [10 - 24] | 0.084 | 11 | [6 - 18] | 13 | [8 - 21] | 0.1 | 12 | [7 - 16] | 14 | [9 - 24] | 0.028 |
| Discharged home | 96 | (63.6) | 26 | (49.1) | 0.064 | 21 | (56.8) | 101 | (60.5) | 0.7 | 64 | (66.0) | 58 | (54.2) | 0.087 |
| Follow-up treatment / rehabilitation | 53 | (35.1) | 24 | (45.2) |  | 16 | (43.2) | 61 | (36.5) |  | 31 | (21.9) | 46 | (43.0) |  |
| In-hospital mortality | 2 | (1.3) | 3 | (5.7) | 0.1 | 0 | (0.0) | 5 | (3.0) | 0.6 | 2 | (2.1) | 3 | (2.8) | 0.9 |

| **Table S4.** Patients cleared by standard approach, but not by the digital-guideline-support (N=116). Values are medians [IQR] or numbers of patients (%). POSPOM, preoperative score to predict postoperative mortality^1^, Charlson Comorbidity index^2^, the surgical risk was determined according to the 2014 ESC guidelines^3^ (30-day risk of cardiovascular death and myocardial infarction^4^) | | |
| --- | --- | --- |
| Biometrics |  |  |
| Age [years] | 73 | [65 - 81] |
| Female sex | 58 | (50.0) |
| Body Mass Index [kg m^-2^] | 26 | [23 - 30] |
| ASA Risk Score |  |  |
| III | 114 | (98.3) |
| IV | 2 | (1.7) |
| Surgical risk |  |  |
| low (<1%) | 10 | (8.6) |
| intermediate (1-5%) | 66 | (56.9) |
| high (>5%) | 40 | (34.5) |
| POSPOM Score | 29 | [26 - 32] |
| Charlson Comorbidity Index |  |  |
| 0 | 22 | (19.0) |
| 1-2 | 64 | (55.2) |
| 3-4 | 15 | (12.9) |
| ≥5 | 2 | (2.0) |
| unknown | 13 | (11.2) |

# References

1 Le Manach Y, Collins G, Rodseth R, et al. Preoperative Score to Predict Postoperative Mortality (POSPOM): Derivation and Validation. *Anesthesiology* 2016; **124**: 570-9

2 D'Hoore W, Sicotte C, Tilquin C. Risk adjustment in outcome assessment: the Charlson comorbidity index. *Methods Inf Med* 1993; **32**: 382-7

3 Kristensen SD, Knuuti J, Saraste A, et al. 2014 ESC/ESA Guidelines on non-cardiac surgery: cardiovascular assessment and management: The Joint Task Force on non-cardiac surgery: cardiovascular assessment and management of the European Society of Cardiology (ESC) and the European Society of Anaesthesiology (ESA). *Eur J Anaesthesiol* 2014; **31**: 517-73

4 Halvorsen S, Mehilli J, Cassese S, et al. 2022 ESC Guidelines on cardiovascular assessment and management of patients undergoing non-cardiac surgery. *Eur Heart J* 2022; **43**: 3826-924
